# Effects of human recombinant granulocyte-colony stimulating factor treatment during *in vitro* culture on porcine pre-implantation embryos

**DOI:** 10.1371/journal.pone.0230247

**Published:** 2020-03-17

**Authors:** Lian Cai, Yeon-woo Jeong, Yong-xun Jin, Jong-yun Lee, Yeon-ik Jeong, Kyu-chan Hwang, Sang-hwan Hyun, Woo-suk Hwang

**Affiliations:** 1 Abu Dhabi Biotech Research Foundation, Seoul, Republic of Korea; 2 College of Veterinary Medicine, Chungbuk National University, Cheongju, South Korea; 3 Department of Animal Science, Chungbuk National University, Cheongju, Chungbuk, Republic of Korea; 4 College of Animal Science, Jilin University, Changchun, China; University of Florida, UNITED STATES

## Abstract

Granulocyte-colony stimulating factor (G-CSF), a pleiotropic cytokine, belongs to the hematopoietic growth factor family. Recent studies have reported that G-CSF is a predictive biomarker of oocyte and embryo developmental competence in humans. The aim of our study was to determine whether *CSF3* and its receptor (*CSF3R*) were expressed in porcine maternal reproductive tissues (oviduct and uterus), cumulus cells, and embryos and to investigate the effects of human recombinant G-CSF (hrG-CSF) supplementation during *in vitro* culture (IVC) on the developmental competence of pre-implantation embryos. To do this, we first performed reverse-transcription polymerase chain reaction (RT-PCR). Second, we performed parthenogenetic activation (PA), *in vitro* fertilization (IVF), and somatic cell nuclear transfer (SCNT) to evaluate the embryonic developmental potential after hrG-CSF supplementation based on various concentrations (0 ng/mL, 10 ng/mL, 50 ng/mL, and 100 ng/mL) and durations (Un-treated, Days 0–3, Days 4–7, and Days 0–7) of IVC. Finally, we examined transcriptional levels of several marker genes in blastocysts. The results of our study showed that *CSF3* transcript was present in all samples we assessed. *CSF3-R* was also detected, except in cumulus cells and blastocysts from PA. Furthermore, 10 ng/mL and Days 0–7 were the optimal concentration and duration for the viability of *in vitro* embryonic development, especially for SCNT-derived embryos. The rate of blastocyst formation and the total cell number of blastocysts were significantly enhanced, while the number and index of apoptotic nuclei were significantly decreased in optimal condition groups compared to others. Moreover, the transcriptional levels of anti-apoptotis- (*BCL2*), proliferation- (*PCNA*), and pluripotency- (*POU5F1*) related genes were dramatically upregulated. In conclusion, for the first time, we demonstrated that *CSF3* and *CSF3R* were expressed in porcine reproductive organs, cells, and embryos. Additionally, we determined that hrG-CSF treatment improved porcine embryonic development capacity *in vitro*.

## Introduction

Owing to their genetic, anatomic, and physiologic similarities with humans [1], porcine animal models are commonly used for studying human diseases, developing novel medical treatment therapies, and xenotransplantation. Recently, xenotransplantation has been widely investigated utilizing interspecies chimeras, created via the injection of human pluripotent stem cells into porcine blastocysts [2]. Due to the associated importance of *in vitro* porcine embryo culture, methods for increasing the viability of embryos are crucial. However, despite extensive research in previous decades, conditions for the production of *in vitro* porcine embryos remain suboptimal [3]. The investigation of maternally-derived factors could aid *in vitro* production methods.

*In vivo*, pre-implantation embryos developed from the zygote to blastocyst stage must traverse the maternal oviduct to the uterus. During this process, numerous maternal growth factors, cytokines, and nutrients provide essential conditions for embryonic development [4–7]. Furthermore, in previous studies, these maternal factors have been shown to affect embryonic development, blastocyst formation rates, blastocyst cell number, metabolism, and apoptosis [4–7].

Granulocyte colony-stimulating factor (G-CSF, CSF3), a pleiotropic cytokine, belongs to the hematopoietic growth factor family, including macrophage-colony stimulating factor (M-CSF, CSF1) and granulocyte macrophage-colony stimulating factor (GM-CSF, CSF2) [8]. Numerous studies have reported that, in several mammalian species, M-CSF [9–11] and GM-CSF [12–15] are expressed in the oviduct and uterus during early pregnancy. Furthermore, they have been implicated in embryonic development. M-CSF enhances the rate of embryonic development [16] and has been shown to increase trophectoderm (TE) cell numbers in mice [10]. GM-CSF has likewise been shown to increase the total cell number of embryos and enhances the capacity of pre-implantation embryonic development in a variety of mammalian species [17–21]. Compared to two such cytokines, reports regarding the roles of G-CSF in reproductive biology are essentially limited to human and mouse. Previous studies have shown that *CSF3* and its receptor (*CSF3R*) were expressed at the fetomaternal interface—including endometrial cells—in murine and human pregnancy [22, 23], and in fallopian tube epithelial cells in humans [24]. However, to the best of our knowledge, no study has surveyed the roles of G-CSF on pre-implantation embryonic developmental competence during *in vitro* culture.

G-CSF is best known for its effects on proliferation, differentiation, and activation of hematopoietic cells of the neutrophilic granulocyte lineage [25, 26]. These effects are reported to be mediated via activation of the Jak/STAT and MAPK pathways [27, 28]. Recently, G-CSF was reported to be involved in the processes of mammalian reproduction. The level of G-CSF in the follicular fluid has been indicated as a predictive biomarker of not only the developmental competence of oocytes and embryos, but also subsequent implantation capacity of embryos after *in vitro* fertilization (IVF)/intracytoplasmic sperm injection (ICSI) in humans [29–32]. Yanagi *et al*. [33] and Salmassi *et al*. [34] demonstrated that G-CSF protein in human follicular fluid was mainly located in granulosa cells and that the expression of G-CSF mRNA in the late follicular phase was ~10 fold higher than that of other phases during the menstrual cycle. G-CSF has been shown to enhance pregnancy and birth rates in patients who suffer from recurrent miscarriage. It also promotes the regeneration of endometrial cells in rats [35–37].

In agreement with the results described above, our previous study showed that *CSF3* and *CSF3R* were also detected in various cells and tissues derived from porcine ovaries such as oocytes, cumulus cells, granulosa cells, and the corpus luteum [38]. We also confirmed that human recombinant G-CSF (hrG-CSF) supplementation during porcine oocyte maturation *in vitro* increased the competence of oocytes to develop as embryos [38]. However, the role of hrG-CSF in *in vitro* pre-implantation embryonic development remains unclear.

The aim of the present study was to determine whether *CSF3* and *CSF3R* are expressed in the porcine reproductive tract and embryos and to investigate the effects of hrG-CSF on pre-implantation embryos during *in vitro* culture (IVC). To do this, we first analyzed the expression of *CSF3* and *CSF3R* mRNA in the oviduct, uterus, cumulus cells, and blastocysts. Second, we performed parthenogenetic activation (PA), IVF, and somatic cell nuclear transfer (SCNT) to evaluate embryonic developmental potential and to count the total and apoptotic cell numbers of blastocysts after hrG-CSF supplementation. Finally, we examined the transcriptional levels of genes selected as markers for proliferation (*PCNA*), pluripotency (*POU5F1*), and apoptosis *(BCL2* and *BAX)* in blastocysts.

## Materials and methods

### Chemicals

Unless otherwise indicated, all chemicals and reagents used in this study were purchased from Sigma-Aldrich Corporation (St. Louis, MO, USA). Given that porcine G-CSF is not currently available, we used human recombinant G-CSF (hrG-CSF, SRP6164; Sigma-Aldrich) in this study. The factor was dissolved in Dulbecco’s phosphate-buffered saline (DPBS; Invitrogen/Thermo Fisher Scientific, Carlsbad, CA, USA) with 0.1% bovine serum albumin (BSA). The final hrG-CSF stock solution was formulated to 10 μg/mL. The control medium consisted of DPBS containing 0.1% BSA. Both solutions were stored at –20°C previous to warming for addition to porcine zygotic medium-3 (PZM-3) [39].

### Oocyte recovery and *in vitro* maturation

Ovaries from prepubertal gilts were collected from a local slaughterhouse and transported to the laboratory within 2 h in physiological saline supplemented with 100 IU/mL penicillin G and 100 mg/mL streptomycin sulfate at 37°C. Follicles 3–6 mm in diameter were aspirated using an 18-gauge needle attached to a 10-mL disposable syringe. Cumulus-oocyte complexes (COCs) were pooled in 15 mL conical tubes and allowed to sediment out of solution at 37°C for 5 min. The supernatant was discarded and the sediment containing the COCs was resuspended with HEPES-buffered Tyrode’s medium (TLH) containing 0.05% (wt/vol) polyvinyl alcohol (TLH-PVA). Oocytes possessing an evenly granulated cytoplasm and 3–10 layers of a compact cumulus mass were selected and washed twice with TLH-PVA. After washing, 50–60 COCs were transferred to a 4-well dish (Nunc, Roskilde, Denmark) with 500 μL *in vitro* maturation (IVM) culture medium (TCM-199; Invitrogen) supplemented with 0.6 mM cysteine, 0.91 mM sodium pyruvate, 10 ng/mL epidermal growth factor (EGF), 75 μg/mL kanamycin, 1 μg/mL insulin, 10% (vol/vol) porcine follicular fluid (pFF), 4 IU/mL equine chronic gonadotropin (eCG), and 4 IU/mL human chronic gonadotropin (hCG; Intervet, Boxmeer, The Netherlands). After 22 h of culture, COCs were transferred to hormone-free IVM medium and cultured for an additional 20–22 h at 39°C under 5% CO_2_ in humidified air. COCs were then denuded by gentle pipetting with 0.1% hyaluronidase and washed three times in TLH-PVA medium. Denuded oocytes with obvious first polar bodies and uniform ooplasm were selected to produce embryos.

### Detection of G-CSF and G-CSF-R by reverse transcription-polymerase chain reaction (RT-PCR) analysis

Gene expression of *CSF* and *CSF3R* was analyzed in porcine oviducts and uteruses in the luteal and follicular phases. Expression levels in cumulus cells and blastocysts derived from *in vitro* fertilization (IVF) and parthenogenetic activation (PA) were also investigated. The follicular phase was defined by follicular maturation and the luteal phase was defined by the presence of the corpus luteum. Uteri and oviducts were collected from a slaughterhouse and mechanically isolated; approximately 0.01 g of the resulting tissue per sample was used for RT-PCR analysis. Tissues were washed three times with PBS containing 0.01% (wt/vol) PVA (PBS-PVA), snap frozen in liquid nitrogen, and ground to a fine powder. Cumulus cells isolated from 120 COCs, 21 IVF blastocysts (Day 7), and 18 PA blastocysts were selected separately under a stereomicroscope. All blastocyst developmental stages (early, expanded, and hatched) from IVF- and PA-derived embryos were used for RT-PCR analysis. Finally, tissues and cells were washed three times with PBS-PVA and transferred to lysis buffer (Dynabeads® mRNA Direct Kit; Dynal Biotech Asa, Oslo, Norway) before snap freezing in liquid nitrogen and storage at –80°C until mRNA isolation could be performed. mRNA was extracted using the Dynabeads® mRNA Direct Kit (Dynal Biotech ASA) followed by routine cDNA synthesis using the LaboPass™ cDNA Synthesis Kit Mastermix (Cosmo Genetech, Seoul, Korea). PCR amplification was performed using 35 cycles, each consisting of 15 sec at 95°C, 15 sec at 58°C, and 30 sec at 72°C. After amplification, 10 μL of the PCR reaction product was electrophoresed on a 1.5% agarose gel and product sizes were verified with a 100-bp DNA ladder (Roche, Mannheim, Germany). The reference gene glyceraldehyde 3-phosphate dehydrogenase (*GAPDH*) was PCR amplified to rule out the possibility of RNA degradation and to control for the variation in mRNA concentrations in the RT reaction.

### Parthenogenetic activation of oocytes

Matured oocytes were rinsed twice in activation medium (280 mM mannitol solution containing 0.001 mM CaCl_2_·2H_2_O and 0.05 mM MgCl_2_·6H_2_O, with pH adjusted to 7.0–7.4 and osmolarity adjusted to 280 mOsm/L). For activation, the oocytes were placed between two electrodes in a chamber containing the activation medium connected to a BTX Electro-cell Manipulator 200 (BTX, Richmond, CA, USA) and subjected to two direct-current (DC) pulses of 380 V/mm for 60 μsec. Activated oocytes were immediately transferred into PZM3 supplemented with 7.5 μg/mL cytochalasin B (CB, C6762) for 3 h. They were then transferred into 30 μL PZM-3 droplets (10 gametes per drop) covered with mineral oil after being washed three times in fresh PZM-3 medium. Embryos were cultured at 39°C in a humidified atmosphere of 5% O_2_, 5% CO_2_, and 90% N_2_ for 168 h (Day 7). In all experiments, the IVC medium was renewed after 48 h (Day 2) and 96 h (Day 4).

### *In vitro* fertilization and culture

IVF and IVC were performed according to a protocol described previously [38]. Briefly, the oocytes were co-incubated with sperm for 20 min at 39°C in a humidified atmosphere of 5% CO_2_ and 95% air. After co-incubation, loosely attached sperm was removed from the zona pellucida (ZP) by gentle pipetting. After washing three times in modified Tris-buffered medium (mTBM) as described previously [40], oocytes were then incubated in fresh mTBM without sperm for 5 to 6 h at 39°C in a humidified atmosphere of 5% CO_2_ and 95% air. Thereafter, fertilized oocytes were cultured in 30 μL micro-drops of PZM-3 (10 ova per drop) after being washed three times with the same medium. Micro-drops were covered with pre-warmed mineral oil and embryos were cultured at 39°C for 168 h (7 days) under a humidified atmosphere of 5% O_2_, 5% CO_2_, and 90% N_2_.

### Micromanipulation for somatic cell nuclear transfer, fusion, and activation

Matured oocytes were incubated for 5 min in manipulation medium (TLH-BSA; calcium-free TLH with 0.2% bovine serum albumin) containing 5 μg/mL Hoechst 33342 (B2261; Sigma-Aldrich) and 5 μg/mL CB. After washing twice in fresh TLH-BSA, oocytes were transferred into a drop of TLH-BSA supplemented with 5 μg/mL CB. Oocytes were enucleated by aspirating their polar bodies and metaphase II chromosome-containing ooplasm using a 16 μm glass pipette (Origio Humagen Pipets, Charlottesville, VA, USA). Enucleation was confirmed by momentary exposure to UV under the control of a shutter system (VCM-D1; Vincent Associates, Rochester, NY, USA). After enucleation, a 14–15 μm trypsinized fetal fibroblast with a smooth cell surface was transferred into the perivitelline space of an enucleated oocyte using a fine injecting pipette. The couplets were washed twice in activation medium (as described for PA) and transferred to a chamber with the fusion medium (260 mM mannitol solution containing 0.1 mM CaCl_2_ and 0.05 mM MgSO_4_). Membrane fusion was induced by two DC pulses of 680 V/mm for 60 μsec. Fused oocytes were washed 3–4 times; membrane fusion was validated using a stereomicroscope after a 30-min incubation in TLH-BSA. Thereafter, SCNT embryos were treated with 2 mM 6-dimethylaminopurine and 0.4 mg/mL demecolcine in the PZM-3 for 4 h under incubation conditions described for PA. Reconstructed embryos were washed three times in fresh PZM-3 medium, transferred into 30 μL PZM-3 droplets covered with pre-warmed mineral oil, and incubated for 7 days.

### Embryo evaluation and total cell count

The day on which PA or IVF was performed was designated Day 0. Cleavage status was checked under a stereomicroscope at 48 h (Day 2). Evenly cleaved embryos were classified into three groups (2 to 3, 4 to 5, and 6 to 8 cells). Blastocyst formation was assessed at 168 h (Day 7) and blastocysts were classified according to their degree of expansion and hatching status as follows: early blastocyst (a small blastocyst with a blastocoel equal to or less than half of the embryo volume), expanded blastocyst (a large blastocyst with a blastocoel greater than half of the embryo volume or a blastocyst with a blastocoel completely filling the embryo), and hatched blastocyst (hatching or already hatched blastocyst) [38]. To determine the total cell number of blastocysts, blastocysts were collected at day 7, washed in PBS-PVA, and fixed in 3.7% paraformaldehyde with PBS-PVA for 10 min before staining with 10 μg/mL Hoechst-33342 for 5 min, followed by a final washing in PBS-PVA. The stained blastocysts were mounted on glass slides in a drop of 100% glycerol, gently covered with a coverslip, observed under a fluorescence microscope (TE300; Nikon, Tokyo, Japan) at 400× magnification, and counted manually.

### Transferase-mediated dUTP nick end labeling (TUNEL) assay

Blastocysts were washed three times in PBS-PVA then fixed in 3.7% paraformaldehyde (w/v) for 1 h at room temperature. After fixation, they were permeabilized with 0.5% Triton X-100 (v/v) for 1 h at 38.5°C. Permeabilized blastocysts were then incubated in the dark for 1 h at 37°C with fluorescein-conjugated deoxyuridine triphosphate (dUTP) and terminal deoxynucleotidyl transferase (Roche, Mannheim, Germany). After nick end labeling, the blastocysts were counterstained with 10 μg/mL Hoechst 33342 for 10 min at room temperature to label nuclei, washed in DPBS-PVA, mounted under a coverslip, and examined under a fluorescence microscope (TE300; Nikon).

### Gene expression analysis by real-time polymerase chain reaction (real-time PCR)

Blastocyst samples (approximately 10 blastocysts per group) were prepared as described above in the RT-PCR analysis section and stored at –80°C for further analysis. Gene expression (Table 1) was analyzed by real-time PCR, ABI PRISM 7300 Sequence Detection System; Applied Biosystems/Thermo Fisher Scientific, Foster City, CA, USA). After mRNA extraction and cDNA synthesis, qRT-PCR reactions were performed using 2 μL of cDNA template with 10 μL SYBR® Master Mixes (Applied Biosystems) containing primers specific to *PCNA*, *POU5F1*, *BCL2*, and *BAX* (Table 1). Reactions were performed for 35 cycles under the following conditions: denaturation at 95°C for 30 sec, annealing at 57°C for 15 sec, and extension at 72°C for 30 sec. Gene expression was quantified relative to the reference gene, *GAPDH*. Relative quantification was based on a comparison of threshold cycle (Ct) at constant fluorescence intensity. Relative mRNA expression (R) was calculated using the following equation: R = 2^-[△Ct sample-△Ct control]^.

**Table 1:** Primer sequences for analysis of mRNA gene expression.

mRNA	Primer sequences (5’-3’)	Product size (bp)	GenBank accession number
*GAPDH*	F: 5’-GTCGGTTGTGGATCTGACCT-3’	207	NM_001206359.1
	R: 5’-TTGACGAAGTGGTCGTTGAG-3’		
*PCNA*	F: 5′-CCTGTGCAAAAGATGGAGTG-3′	187	XM_003359883
	R: 5′-TTTTCGGTGAGGTGAGAGAGG-3′		
*POU5F1*	F: 5′-GCGGACAAGTATCGAGAACC-3′	200	NM_001113060
	R: 5′-CGTTGCTCTCCTAAAACTCC-3′		
*BCL2*	F: 5’-AATGACCACCTAGAGCCTTG-3’	182	NM_214285
	R: 5’-GGTCATTTCCGACTGAAGAG-3’		
*BAX*	F: 5’-TGCCTCAGGATGCATCTACC-3’	199	XM_003127290
	R: 5’-AAGTAGAAAAGCGCGACCAC-3’		
*CSF3*	F: 5’- GAGCTTCCTGGAGCTGGCGTAC-3’	208	NM213842
	R: 5’-TGCTACAGGCGGGAGAAT-3		
*CSF3R*	F: 5’-CTGGGCCTGCTTCTTGATAA-3’	202	XM_021095950.1
	R: 5’-GGCTAGTGGACAGGTCTGGA-3		

F: Forward, R: Reverse.

### Statistical analysis

Each experiment consisted of at least three replicates; for each replicate, oocytes were collected on the same day from the same group of slaughterhouse-derived ovaries. Statistical analysis was carried out using SPSS 17.0 (SPSS, Inc., Chicago, IL, USA). Embryonic development (e.g., rates of cleavage and blastocyst formation) was analyzed by the chi-squared test. The total cell number of blastocysts derived from PA and IVF were compared by one-way analysis of variance (ANOVA), followed by Duncan’s multiple range tests. T-tests were conducted to assess the apoptotic differences between cloned blastocysts and for relative gene expression levels. All data are presented as mean ± SEM. *P* < 0.05 was considered significant.

## Results

### Detection of G-CSF and its receptor by RT-PCR

**Fig 1:**
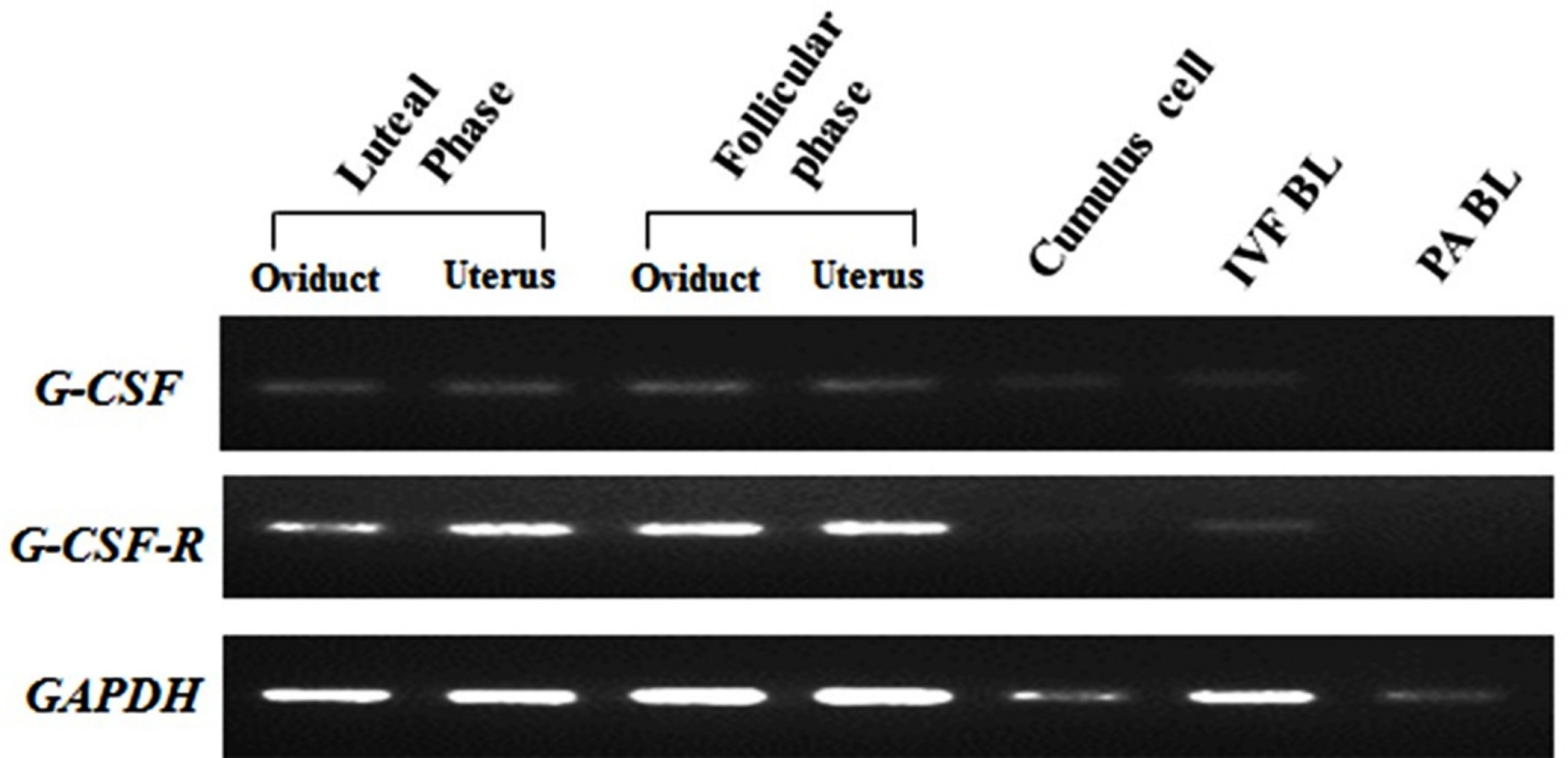
Detection of CSF3 and CSF3R by RT-PCR. mRNA expression of *CSF3* and *CSF3R* in porcine oviduct and uterus derived from follicular phase and luteal phase, as well as cumulus cells and blastocysts derived from IVF and PA by reverse transcription-PCR and agarose gel electrophoresis.

The results showed that amplicons were detectable in samples from IVF-derived blastocysts, as well as in the oviduct and uterus, respectively, derived from both phases. On the contrary, almost no expression of *CSF3R* was detected in cumulus cells and parthenogenetic blastocysts expressed neither *CSF3* nor its receptor (Fig 1).

### Effects of hrG-CSF supplementation at various concentrations on porcine *in vitro* development of PA and IVF embryos

To identify the optimal concentration of hrG-CSF for the embryonic development of porcine parthenotes and IVF-derived embryos, embryos were cultured in IVC medium containing 0, 10, 50, and 100 ng/mL hrG-CSF as previously described [38] for 168 h (7 days). The embryos were evaluated for cleavage and blastocyst presence after 48 h (2 days) and 168 h (7 days), respectively, using a stereomicroscope. hrG-CSF had no significant effect on the cleavage rate or blastocyst formation rate in PA-derived embryos. Blastocyst total cell numbers were, however, slightly increased in the 50 ng/mL hrG-CSF treatment group compared to untreated controls (49.2 ± 1.0, n = 41 and 44.0 ± 1.1, n = 36, respectively; Table 2). In IVF-derived embryos, blastocyst formation was enhanced in the 10 ng/mL hrG-CSF treated group (28.7%) compared to that in the 100 ng/mL hrG-CSF treated and non-treated groups (16.9% and 19.3%, respectively; Table 3). Furthermore, total cell number was significantly higher in embryos treated with 10 ng/mL hrG-CSF (62.5 ± 4.5) compared to those treated with 100 ng/mL hrG-CSF (41. 0 ± 2.9) and those that were non-treated (44.3 ± 3.0; Table 3). The effective optimal concentration of hrG-CSF was consistent with levels previously reported [38].

**Table 2:** Effects of hrG-CSF supplementation at various concentrations on porcine *in vitro* development of parthenotes.

hrG-CSF Concentration (ng/mL)	No. of oocytes Cultured (4)*	No. (%) of embryos developed to		Blastocyst cell Number (N)
		Cleaved (%)^†^	Blastocyst (%)^†^	
**0**	144	127 (88.5)	46 (31.8)	44.0 ± 1.1(26)^a^
**10**	142	126 (88.4)	50 (35.0)	48.1 ± 1.0(31)^bc^
**50**	158	143 (90.2)	45 (28.2)	49.2 ± 1.0(25)^c^
**100**	141	125 (88.8)	46 (32.5)	44.9 ± 1.2(24)^ab^

Values with different superscripts within the same column are significantly different (*P* < 0.05).

The data represent means ± SEM.

* Number of replicates.

N Number of blastocysts examined.

† Percentage of total cultured oocytes.

**Table 3:** Effects of hrG-CSF supplementation at various concentrations on porcine *pre-implantation* embryonic development of *in vitro* fertilization embryos.

hrG-CSF Concentration (ng/mL)	No. of oocytes Cultured (3) *	No. (%) of embryos developed to		Blastocyst cell Number (N)
		Cleaved (%) ^†^	Blastocyst (%) ^†^	
**0**	88	62(70.5)	17(19.3)	44.3 ± 3.0(17)^ab^
**10**	87	57(65.5)	25(28.7)	62.5 ±4.5 (23)^c^
**50**	91	58(63.7)	21(23.1)	58.4 ± 6.9(21)^bc^
**100**	83	52(62.7)	14(16.9)	41.0 ± 2.9(14)^a^

Values with different superscripts within the same column are significantly different (*P* < 0.05).

The data represent means ± SEM.

* Number of replicates.

N Number of blastocysts examined.

† Percentage of total cultured oocytes.

### Effects of hrG-CSF treatment on *in vitro* development of PA and IVF embryos according to the duration of treatment

We established four groups for PA- and IVF-derived embryos according to the duration of 10 ng/mL hrG-CSF supplementation. These four groups included the non-treated control and treatment during the first 89 h (Days 0–3), the second 89 h (Days 4–7), and the entire 178 h (Days 0–7), respectively. No difference in parthenote development capacity was observed between hrG-CSF treatment groups. Nevertheless, blastocyst cell numbers were significantly increased in the 178 h-treated embryos (51.2 ± 2.1) compared to the first 89 h treated and non-treated embryos (45.3 ± 1.7 and 42.0 ± 1.5, respectively; Table 4). In IVF-derived embryos, the entire 178 h treatment group yielded increased blastocyst formation rate (22.0%) compared to the other three groups (14.8%, 17.7%, and 18.3%; Table 5). However, there was no observed effect on cell numbers in IVF blastocysts.

**Table 4:** Effects of hrG-CSF (10 ng/mL) treatment duration on *in vitro* development of parthenotes.

Groups	No. of oocytes	No. (%) of embryos developed to		Blastocyst cell Number (N)
	cultured (3)*	≥ 2-cells (%)^†^	Blastocysts (%) ^†^	
**control**	89	66(74.1)	29(32.8)	42.0 ± 1.5(28)^a^
**Day 0 to 3**	89	66(74.2)	30(33.9)	45.3 ± 1.7(29)^a^
**Day 4 to 7**	89	69(77.6)	28(31.4)	46.4 ± 1.9(28)^a,b^
**Day 0 to 7**	89	65(73.2)	31(34.9)	51.2 ± 2.1(31)^b^

Values with different superscripts within the same column are significantly different (*P* < 0.05).

The data represent means ± SEM.

hrG-CSF: human recombinant granulocyte-colony stimulating factor.

* Number of replicates.

N Number of blastocysts examined.

† Percentage of total cultured oocytes.

**Table 5:** Effects of hrG-CSF (10 ng/mL) treatment duration on *in vitro* development of *in vitro* fertilization embryos.

Groups	No. of oocytes	No. (%) of embryos developed to		Blastocyst cell Number (N)
	cultured (3)*	≥ 2-cells (%)^†^	Blastocysts (%) ^†^	
**control**	81	57 (70.4)	12 (14.8)	54.8 ± 2.0(12)
**Day 0 to 3**	85	52 (61.3)	15 (17.7)	54.7 ± 1.8(15)
**Day 4 to 7**	82	53 (64.7)	15 (18.3)	52.1 ± 2.2(15)
**Day 0 to 7**	82	56 (68.4)	18 (22.0)	54.4 ± 0.3(18)

Values with different superscripts within the same column are significantly different (*P* < 0.05).

The data represent means ±SEM.

hrG-CSF: human recombinant granulocyte-colony stimulating factor.

* Number of replicates.

N Number of blastocysts examined.

† Percentage of total cultured oocytes.

### Effect of uninterrupted hrG-CSF treatment on the *in vitro* development of cloned embryos

Supplementation of hrG-CSF for the entire IVC period increased the percentage of cloned embryo blastocyst formation compared to non-treated embryos (24.6% vs. 18.0%, respectively; Table 6).

**Table 6:** Effects of hrG-CSF treatment for entire stage culture duration on *in vitro* development of cloned embryos.

Groups	No. of oocytes	No. (%) of embryos developed to	
	cultured (9)*	≥ 2-cells (%) ^†^	Blastocysts (%) ^†^
Control	259	181(71.06)	48(18.04) ^a^
10 ng/mL	258	185(72.77)	63(24.60) ^b^

Values with different superscripts within the same column are significantly different (*P* = 0.103).

The data represent means ± SEM.

hrG-CSF: human recombinant granulocyte-colony stimulating factor.

* Number of replicates.

N Number of blastocysts examined.

† Percentage of total cultured oocytes.

### Effects of hrG-CSF treatment on total cell number and apoptosis in SCNT blastocysts

**Fig 2:**
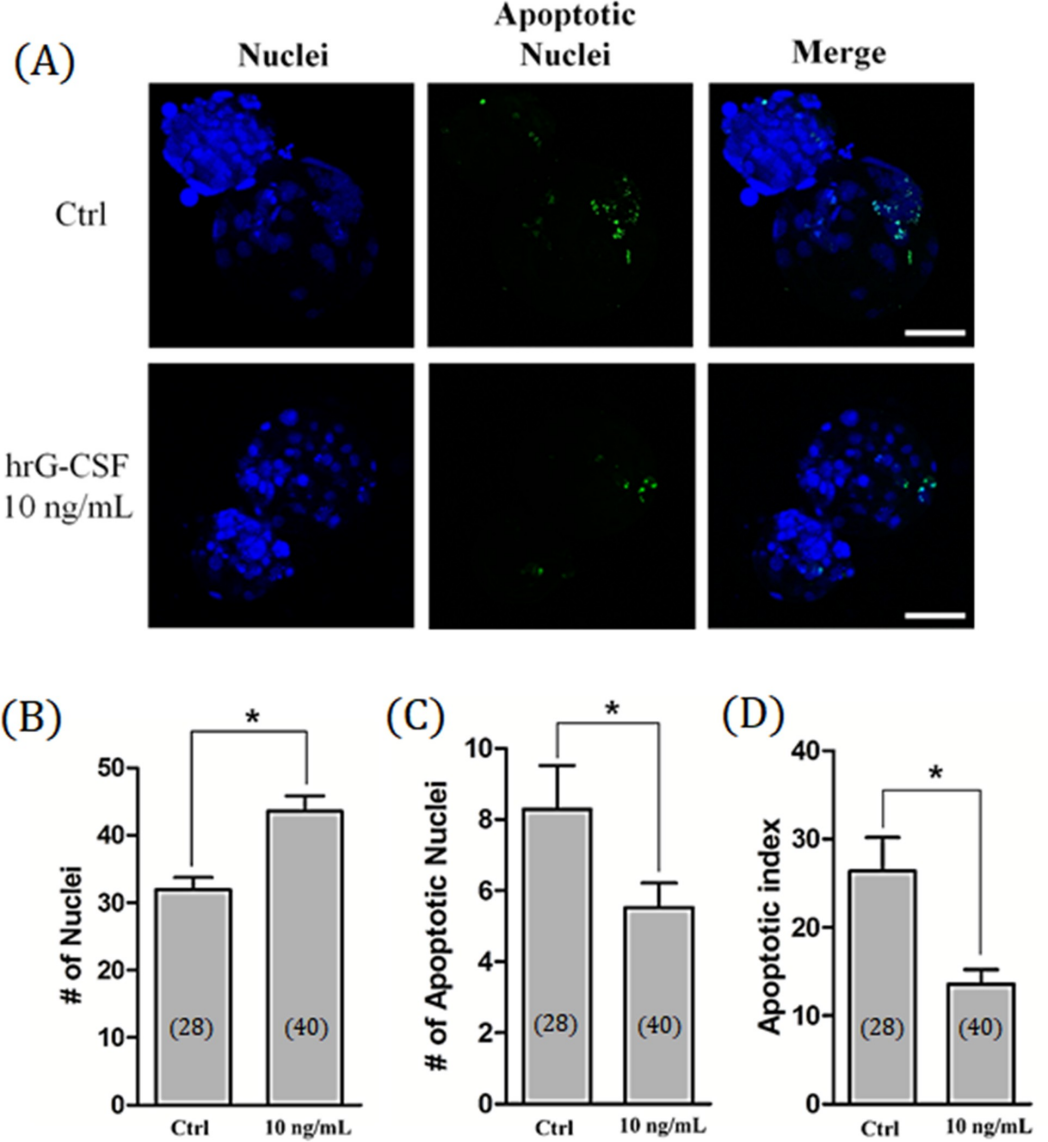
Total cell number and apoptosis of cloned blastocyst after hrG-CSF treatment. Representative laser scanning confocal microscopy images (400×) of nuclei (blue) and fragmented DNA (green) in porcine blastocysts after culturing for 7 days with (10 ng/mL) or without (Control) hrG-CSF treatment. Scale bar = 100 μm (A). The total cell number of nuclei (B), apoptotic nuclei (C), and apoptotic index (D) in porcine cloned blastocysts developed *in vitro*. Ctrl: no treatment; hrG-CSF 10 ng/mL: human recombinant granulocyte-colony stimulating factor 10 ng/mL treatment for entire stage (Days 0 to 7). The number of embryos examined in each experimental group is shown in parentheses. *: *P* < 0.05.

To examine the quality of cloned porcine blastocysts, the total number of nuclei and incidence of apoptosis were counted. The total number of nuclei was significantly (*P* < 0.05) enhanced in embryos treated with hrG-CSF compared to non-treated embryos (43.56 ± 2.3 vs. 31.9 ± 1.8, respectively; Fig 2A and 2B). Furthermore, the number of apoptotic nuclei and the apoptotic index was significantly decreased in embryos treated with hrG-CSF compared to non-treated controls (5.5 ± 0.7 vs. 8.3 ± 1.2 and 13.6% vs. 26.4%, respectively; Fig 2A, 2C and 2D).

### Effects of hrG-CSF treatment on mRNA expression levels of PA, IVF, and cloned blastocysts

**Fig 3:**
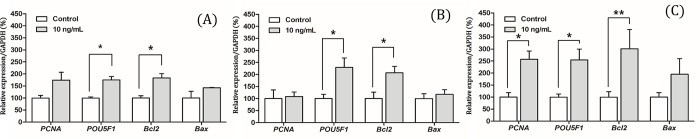
Analysis of gene expression levels in porcine Day 7 blastocysts. The transcriptional levels of *PCNA*, *POU5F1*, *BCL2*, and *BAX* genes were analyzed in porcine blastocysts derived from parthenogenetic activation (A), *in vitro* fertilization (B), somatic cell nuclear transfer (C) by real-time PCR. Data are means ± SEM. *P < 0.05, ** *P* = 0.0709.

Expression levels of *PCNA*, *POU5F1*, *BCL2*, and *BAX* were evaluated in PA, IVF, and SCNT-derived blastocysts. *POU5F1* and *BCL2* transcripts were significantly higher in hrG-CSF-treated than in non-treated blastocysts in all groups (Fig 3). *PCNA* mRNA transcript levels were significantly increased only in SCNT-derived hrG-CSF-treated blastocysts (Fig 3C).

### Discussion

Here we show for the first time that *CSF3* and *CSF3R* are expressed in porcine reproductive organs, cumulus cells, and blastocysts of the porcine reproductive system. Furthermore, the blastocysts derived from IVF expressed *CSF3R*. We had speculated that G-CSF was one of the maternally-derived factors needed for embryonic development. We found that hrG-CSF supplementation increased the capacity of embryonic development the total cell number of blastocysts and decreased the number of apoptotic nuclei with a corresponding upregulation of the expression of genes related to proliferation (*PCNA*), pluripotency (*POU5F1*), and anti-apoptosis (*BCL2*).

In previous studies, M-CSF and GM-CSF have been reported to increase murine, bovine, and porcine blastocyst formation in *in vitro* culture conditions [16, 18, 20]. Furthermore, GM-CSF has been shown to increase cell numbers of both the inner cell mass (ICM) and trophectoderm and reduce apoptotic nuclei in humans [41]. Moreover, it improved the hatch rate of embryos from their zona pellucida and the attachment rate of embryos in *in vitro* culture in humans [17] and mice [18]. The effects of G-CSF on the potential of pre-implantation embryonic development remain unclear. We speculated that G-CSF may play a role similar to other members of the CSF family. In the present study, we investigated the effects on the developmental potential of PA, IVF, and SCNT embryos. Interestingly, it was found that the most prominent effect of hrG-CSF was on cloned embryos. In contrast, there was no improvement in the proportion of PA blastocyst formation and no expression of *CSF3R* in PA blastocysts. However, transcript levels of *POU5F1* and *BCL2* were found to be increased in all three types of hrG-CSF-treated blastocysts. These findings may be due to the expression of *CSF3R* in porcine oocytes, as we described previously [38]; alternatively, the receptor may be temporarily expressed during the early stages of embryonic development, which we did not evaluate here. Another possible explanation for these results might involve the difference in global levels of epigenetic reprogramming in porcine *in vitro-*derived embryos (PA, IVF, and SCNT) such as DNA methylation [42], leading to the observed differences in the effects of hrG-CSF. Taken together, the present findings are consistent with previous reports on the other two members of the CSF family [16–18, 20, 41].

Considering previous reports, these molecules may be produced during specific periods of pre-implantation embryonic development. An example of such would be interleukin-1β, which is thought to exert its action on embryonic growth before Day 5 in bovines [43]. GM-CSF has been reported to increase the cell number of blastocysts and the proportion of blastocysts formed after Day 4 (when embryos were at the morula stage of development) in porcine and bovine models [19, 23]. As a result of this temporal difference and in order to establish a more exact action period, hrG-CSF was added to cultures during the first 89 h (Days 0–3), the second 89 h (Days 4–7), and the entire 179 h (Days 0–7) of embryonic culture. Interestingly, our current study demonstrated that hrG-CSF supplementation increased the ratio of IVF embryos that became blastocysts, regardless of the treatment period. The continuously treated group showed the highest proportion of blastocyst stage embryos. This may be related to the *CSF3* expression found in both follicular and luteal phase oviducts and uteri as well as cumulus cells.

Defects in DNA modification and chromosome remodeling have been detected frequently in embryos cultured *in vitro*, especially those derived from SCNT [42, 44]. These complicated defects in pre-implantation embryos may lead to a series of additional defects, such as aberrant expression of *POU5F1*, which is important for establishing the distinct identity of the ICM in early embryogenesis [45, 46]; they also have a role in the control of embryonic developmental pluripotency [47]. A previous study reported that *POU5F1* stealth siRNA-injected porcine embryos were arrested at 8 cell stage [48] and the embryos that expressed a low level of this gene lost pluripotency and were nearly exclusively differentiated into the TE [47]. Nuclear reprogramming involves resetting the epigenetic mechanisms that maintain stable gene expression—for instance, DNA methylation and histone modifications [49]. Incomplete reprogramming in embryos derived from SCNT may lead to the abnormal development of cloned embryos [50]. One candidate is the incomplete reactivation of *POU5F1* and several *POU5F1*-related genes [51]. In this study, we observed an increased expression of *POU5F1* in hrG-CSF-treated blastocysts. Overall, we speculated that hrG-CSF may overcome some of the reprogramming deficiencies and plays a positive role in the maintenance of sufficient pluripotency during porcine pre-implantation embryonic development.

Embryos cultured *in vitro* can be affected by environmental stressors that are potential inducers of unplanned apoptosis. This apoptosis may lead to embryo arrest or influence embryo viability [52]. *BCL2* is associated with anti-apoptotic effect and has been primarily detected after embryonic genome activation [53]. In bovines, healthy pre-implantation embryos have been reported to express high levels of *BCL2*, while fragmented embryos exhibit low expression levels [54]. In our investigation, hrG-CSF enhanced *BCL2* expression levels in PA, IVF, and SCNT-derived blastocysts. Accordingly, our study indicated that hrG-CSF may have anti-apoptotic effects.

The role of G-CSF on proliferation according to different cell types remains controversial. Miyamay *et al*. suggested that G-CSF plays a role in promoting the proliferation of trophoblast cells [55]. G-CSF has been shown to have different roles in various cell types; for instance, Kumar *et al*. showed that it does not affect the proliferation of ovarian cancer cell lines but does protect against apoptosis [56]. *PCNA* is an essential component of the DNA replication and repair machinery [57] and has been used as a proliferative marker during bovine embryonic development [58]. Our findings suggested that the expression of *PCNA* is only significantly enhanced in hrG-CSF-treated SCNT embryos, not in PA and IVF embryos. This is in-line with previous reports that embryos originating from different reproductive technologies display distinguishable global gene expression [42, 59]. Therefore, hrG-CSF exhibited different proliferation effects on these three types of *in vitro* blastocysts that are shown not only in the total cell number of blastocysts but also in the transcription level of *PCNA*.

In conclusion, we found that hrG-CSF enhanced porcine pre-implantation embryonic developmental competence *in vitro*. In other words, G-CSF may play a role in porcine embryonic development by maintaining embryo pluripotency and exhibiting anti-apoptotic and proliferative effects. G-CSF is likely a maternal factor secreted by maternal reproductive tissues.

## Supporting information

S1 File (XLSX)
